# Faulty cardiac repolarization reserve in alternating hemiplegia of childhood broadens the phenotype

**DOI:** 10.1093/brain/awv243

**Published:** 2015-08-21

**Authors:** Fatima Jaffer, Andreja Avbersek, Rosaria Vavassori, Carmen Fons, Jaume Campistol, Michela Stagnaro, Elisa De Grandis, Edvige Veneselli, Hendrik Rosewich, Melania Gianotta, Claudio Zucca, Francesca Ragona, Tiziana Granata, Nardo Nardocci, Mohamed Mikati, Ashley R. Helseth, Cyrus Boelman, Berge A. Minassian, Sophia Johns, Sarah I. Garry, Ingrid E. Scheffer, Isabelle Gourfinkel-An, Ines Carrilho, Sarah E. Aylett, Matthew Parton, Michael G. Hanna, Henry Houlden, Brian Neville, Manju A. Kurian, Jan Novy, Josemir W. Sander, Pier D. Lambiase, Elijah R. Behr, Tsveta Schyns, Alexis Arzimanoglou, J. Helen Cross, Juan P. Kaski, Sanjay M. Sisodiya

**Affiliations:** 1 1 MRC Centre for Neuromuscular Diseases, The National Hospital for Neurology and Neurosurgery, Queen Square, London, WC1N 3BG, UK; 2 2 Department of Molecular Neuroscience, UCL Institute of Neurology, Queen Square, London, WC1N 3BG, UK; 3 3 NIHR UCLH Biomedical Research Centre Department of Clinical and Experimental Epilepsy, UCL Institute of Neurology, Queen Square, London, WC1N 3BG, UK; 4 4 Epilepsy Society, Chalfont-St-Peter, Bucks, SL9 0RJ, UK; 5 5 A.I.S.EA Onlus, Via Sernovella, 37 - Verderio Superiore, 23878 Lecco, Italy; 6 6 Paediatric Neurology Department, Hospital Sant Joan de Déu, P° de Sant Joan de Déu, 2 08950 Esplugues de Llobregat, Barcelona University, Barcelona, Spain; 7 7 Child Neuropsychiatry Unit, Istituto Giannina Gaslini, Department of Neurosciences, Rehabilitation, Ophthalmology, Genetics and Maternal and Children's Sciences, Istituto Giannina Gaslini, Largo Gaslini 5, 26148, University of Genoa, Genoa, Italy; 8 8 University Medical Center Göttingen, Georg August University, Department of Pediatrics and Adolescent Medicine, Division of Pediatric Neurology, Georg August University, Robert Koch Strasse 40, 37099 Göttingen, Germany; 9 9 Child Neurology Unit IRCCS Istituto delle Scienze Neurologiche di Bologna, Ospedale Bellaria, Via Altura 3, 40139 Bologna, Italy; 10 10 Clinical Neurophysiology Unit, IRCCS “E. Medea”, Via Don L. Monza 20, 23842 Bosisio Parini (LC), Italy; 11 11 Department of Pediatric Neuroscience, IRCCS Foundation Neurological Institute C. Besta, Via Celoria 11, 20133 Milano, Italy; 12 12 Division of Paediatric Neurology, Duke University, T0913J Children Health Centre, Duke University Medical Centre, Durham, USA; 13 13 Division of Neurology, Department of Paediatrics, The Hospital for Sick Children and University of Toronto, 555 University Avenue, Toronto, Ontario, Canada, M5G 1X8; 14 14 Inherited Cardiovascular Diseases Unit, Great Ormond Street Hospital for Children NHS Foundation Trust, and Institute of Cardiovascular Science, University College London, London, WC1N 3JH, UK; 15 15 Florey Institute of Neurosciences and Mental Health, and Department of Paediatrics, University of Melbourne, Royal Children’s Hospital, Melbourne, Australia; 16 16 Centre de reference epilepsies rares et Sclérose tubéreuse de Bourneville (site Parisien adolescents-adultes), Hôpital Pitié-Salpêtrière, 47-83, boulevard de l’Hôpital 75651 Paris cedex 13, France; 17 17 Neuropediatric Department Centro Hospitalar do Porto, Rua da Boavista, 8274050-111, Porto, Portugal; 18 18 Clinical Neurosciences, Developmental Neuroscience Programme, UCL Institute of Child Health, & Great Ormond Street Hospital for Children NHS Foundation Trust, London, WC1N 3JH, UK; 19 19 Molecular Neurosciences, Developmental Neurosciences Programme, UCL Institute of Child Health and Department of Neurology, Great Ormond Street Hospital, London, London, WC1N 3JH, UK; 20 20 Department of Cardiac Electrophysiology, The Heart Hospital, Institute of Cardiovascular Science, University College London, 16-18 Westmoreland St, London W1G 8PH, UK; 21 21 Cardiac and Cell Sciences Institute, St George’s University of London, Cranmer Terrace, London SW17 0RE, UK; 22 22 European Network for Research on Alternating Hemiplegia, ENRAH, Brussels, Belgium; 23 23 Epilepsy, Sleep and Paediatric Neurophysiology Department (ESEFNP), University Hospitals of Lyon (HCL), and DYCOG team, Lyon Neuroscience Research Centre (CRNL), INSERM U1028; CNRS UMR 5292, Lyon, France; 24 24 Young Epilepsy, St. Piers Lane, Lingfield, Surrey RH7 6PW, UK

**Keywords:** alternating hemiplegia of childhood, *ATP1A3*, Na^+^/K^+^-ATPase, SUDEP, electrocardiogram

## Abstract

Alternating hemiplegia of childhood is a rare disorder caused by *de novo* mutations in the *ATP1A3* gene, expressed in neurons and cardiomyocytes. As affected individuals may survive into adulthood, we use the term ‘alternating hemiplegia’. The disorder is characterized by early-onset, recurrent, often alternating, hemiplegic episodes; seizures and non-paroxysmal neurological features also occur. Dysautonomia may occur during hemiplegia or in isolation. Premature mortality can occur in this patient group and is not fully explained. Preventable cardiorespiratory arrest from underlying cardiac dysrhythmia may be a cause. We analysed ECG recordings of 52 patients with alternating hemiplegia from nine countries: all had whole-exome, whole-genome, or direct Sanger sequencing of *ATP1A3.* Data on autonomic dysfunction, cardiac symptoms, medication, and family history of cardiac disease or sudden death were collected. All had 12-lead electrocardiogram recordings available for cardiac axis, cardiac interval, repolarization pattern, and J-point analysis. Where available, historical and prolonged single-lead electrocardiogram recordings during electrocardiogram-videotelemetry were analysed. Half the cohort (26/52) had resting 12-lead electrocardiogram abnormalities: 25/26 had repolarization (T wave) abnormalities. These abnormalities were significantly more common in people with alternating hemiplegia than in an age-matched disease control group of 52 people with epilepsy. The average corrected QT interval was significantly shorter in people with alternating hemiplegia than in the disease control group. J wave or J-point changes were seen in six people with alternating hemiplegia. Over half the affected cohort (28/52) had intraventricular conduction delay, or incomplete right bundle branch block, a much higher proportion than in the normal population or disease control cohort (*P = *0.0164). Abnormalities in alternating hemiplegia were more common in those ≥16 years old, compared with those <16 (*P = *0.0095), even with a specific mutation (p.D801N; *P = *0.045). Dynamic, beat-to-beat or electrocardiogram-to-electrocardiogram, changes were noted, suggesting the prevalence of abnormalities was underestimated. Electrocardiogram changes occurred independently of seizures or plegic episodes. Electrocardiogram abnormalities are common in alternating hemiplegia, have characteristics reflecting those of inherited cardiac channelopathies and most likely amount to impaired repolarization reserve. The dynamic electrocardiogram and neurological features point to periodic systemic decompensation in *ATP1A3*-expressing organs. Cardiac dysfunction may account for some of the unexplained premature mortality of alternating hemiplegia. Systematic cardiac investigation is warranted in alternating hemiplegia of childhood, as cardiac arrhythmic morbidity and mortality are potentially preventable.

## Abstract (precis)

Alternating hemiplegia of childhood is rare and usually results from mutations in cardiac- and brain-expressed *ATP1A3.* In an ECG study of 52 cases, Jaffer *et al.* reveal dynamic cardiac repolarisation or conduction abnormalities in over 50%. Abnormalities are more common in those ≥16 years, and suggest impaired cardiac repolarisation reserve.

## Graphical abstract

**Figure:**
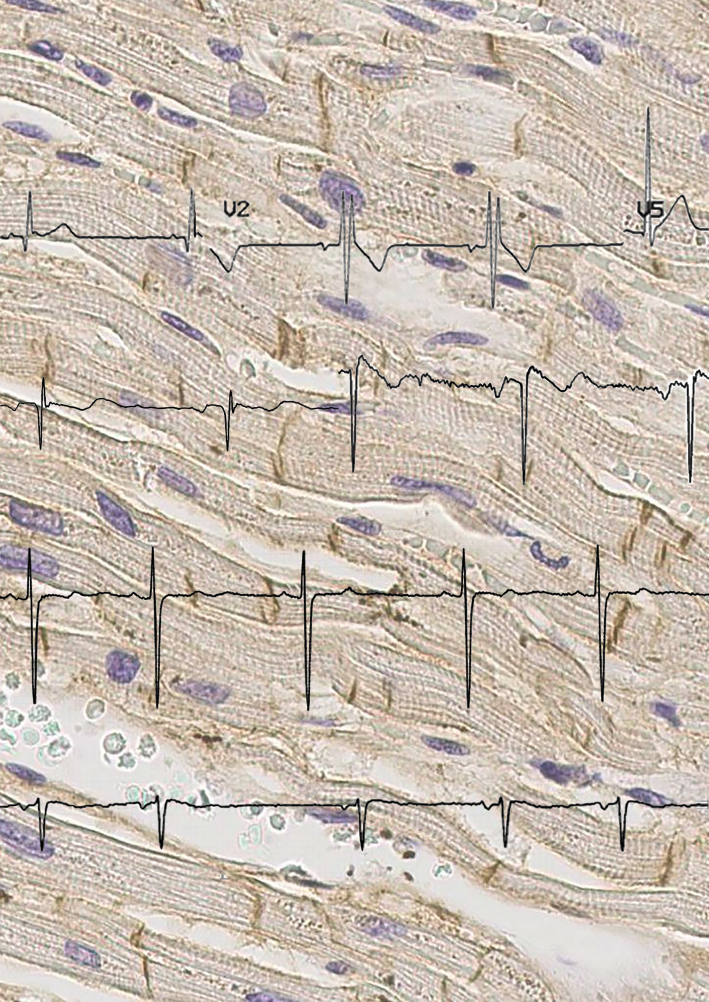
Alternating hemiplegia of childhood is rare and usually results from mutations in cardiac- and brain-expressed *ATP1A3.* In an ECG study of 52 cases, Jaffer *et al.* reveal dynamic cardiac repolarisation or conduction abnormalities in over 50%. Abnormalities are more common in those ≥16 years, and suggest impaired cardiac repolarisation reserve.

## Introduction

Alternating hemiplegia of childhood (OMIM #104290) is a rare neurodevelopmental disorder with onset before the age of 18 months and prevalence estimated at 1:1 000 000 to 1:100 000 (Neville and Ninan, 2007; Gilissen *et al.*, 2012). Affected children typically survive to adulthood, and we use the label ‘alternating hemiplegia’. Pathogenic mutations, almost always *de novo*, in the *ATP1A3* gene, encoding the catalytic alpha-3 subunit of the Na^+^/K^+^-ATPase transporter protein, are the cause in ∼80% of cases (Heinzen *et al.*, 2012; Rosewich *et al.*, 2012; Ishii *et al.*, 2013). No other cause is known.

Alternating hemiplegia is characterized by recurrent transient plegic or paretic attacks, affecting alternate or both sides of the body, dystonic posturing, and oculomotor dysfunction (Bourgeois *et al.*, 1993; Aicardi *et al.*, 1995; Panagiotakaki *et al.*, 2010). Seizures are common, as are non-paroxysmal features including: dystonia, choreoathetosis, ataxia, pyramidal signs, developmental delay and varying degrees of intellectual disability. Dysautonomia, manifesting as dyspnoea, stridor, apnoea, pallor, fever, and altered heart rate, is frequently described during plegic episodes. Occasionally, autonomic dysfunction can occur in isolation (Panagiotakaki *et al.*, 2010). Recently, asystole associated with new-onset episodes of collapse with loss of consciousness, cyanosis and respiratory arrest was reported in a patient with genetically-confirmed alternating hemiplegia, benefitting from implantation of a permanent pacemaker (Novy *et al.*, 2014).

Cardiac channelopathies, such as long QT syndrome, Brugada syndrome, and catecholaminergic polymorphic ventricular tachycardia, are associated with an increased risk of malignant arrhythmias and sudden cardiac death (Wilde *et al.*, 2013). Most of the causative genes are expressed in a number of tissues, and neuromuscular manifestations are increasingly recognized (Abriel *et al.*, 2013). Some neuronal channelopathies, such as the multisystem disorder Andersen-Tawil syndrome, associated with mutations in the *KCNJ2* gene, which is expressed in the brain and heart, can also cause long QT syndrome (type 7; OMIM #170390), increasing the risk of sudden cardiac death; these patients are routinely kept under cardiac surveillance. Patients with Dravet syndrome (OMIM #607208) also have an elevated risk of premature mortality, ascribed largely to sudden unexpected death in epilepsy (SUDEP) (Hindocha *et al.*, 2008; Genton *et al.*, 2011). Some individuals with Dravet syndrome exhibit reduced heart rate variability; ECG recordings may show increased P-wave and QT dispersion, possibly contributing to mechanisms of sudden death in Dravet syndrome (Delogu *et al.*, 2011; Ergul *et al.*, 2013). Other syndromes with mutations in ion-channel genes expressed in the brain and the heart, termed ‘cardiocerebral channelopathies’ have features related to both organs and may also cause sudden death (Heron *et al.*, 2010; Parisi *et al.*, 2013).

Other than altered heart rate and a single report of asystole, cardiac abnormalities have not been extensively described in alternating hemiplegia, but sudden unexplained death has been reported (Panagiotakaki *et al.*, 2010; Novy *et al.*, 2014). *ATP1A3* is known to be expressed in the human and rat heart (Zahler *et al.*, 1993; Aye *et al.*, 2010). We hypothesized that important electrocardiographic abnormalities are present in alternating hemiplegia.

## Materials and methods

### Participants

This research was approved by local ethics committees of the participating centres: The National Hospital for Neurology and Neurosurgery UK; Great Ormond Street Hospital for Children UK; Hospital Sant Joan de Déu Barcelona, Spain; Istituto Giannina Gaslini, University of Genoa, Italy; University Medical Center Göttingen, Germany; C.Besta Neurological Institute Milan, Italy; IRCCS E.Medea, Italy; Duke University Medical Center, Durham, USA; The Hospital for Sick Children and University of Toronto, Toronto, Canada; Royal Children’s Hospital Melbourne, Australia; Hôpital Pitié-Salpêtrière, Paris, France; and Neuropediatric Department, Hospital Maria Pia do Centro Hospitalar do Porto, Portugal.

Informed consent was obtained from patients or their parents, or legal guardians in the case of minors or those with intellectual disability.

**Figure 1:**
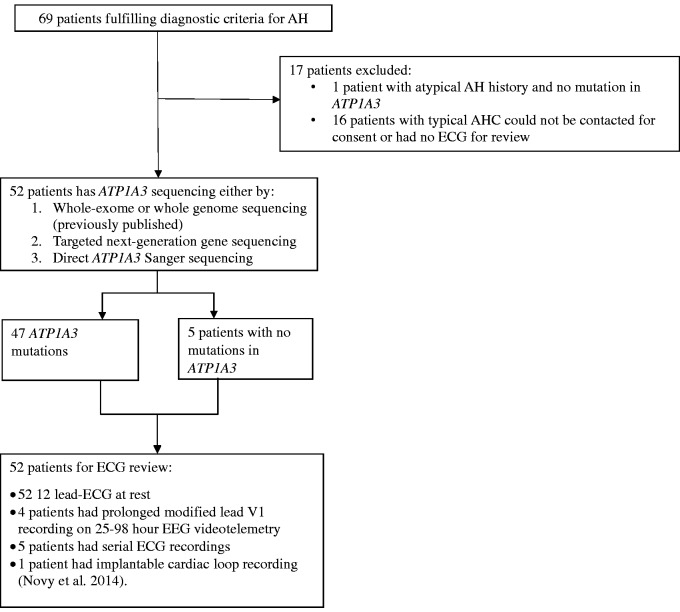
**Study profile of patients recruited into study of ECG characteristics in patients with alternating hemiplegia.** AH = alternating hemiplegia; AHC = alternating hemiplegia of childhood.

Participants were recruited through the International Alternating Hemiplegia of Childhood Research Consortium (IAHCRC), and the European Network for Research on Alternating Hemiplegia (ENRAH), or personal communication with collaborators, from nine countries. A total of 69 patients meeting the clinical diagnostic criteria for typical alternating hemiplegia were identified: 52 were suitable for inclusion (Aicardi *et al.*, 1995; Panagiotakaki *et al.*, 2010). Patients were excluded if they could not be consented or DNA could not be obtained for *ATP1A3* testing if previous mutation analysis had not been undertaken (Fig. 1), or an ECG recording was unavailable.

We collected 52 fully anonymized ECGs from disease controls, all of whom had epilepsy, and ranged in age from 1 month to 36 years. Demographics and details on ECG findings, epilepsy and treatments for the disease controls are provided in Supplementary Table 2.

### Procedures

Clinical data about alternating hemiplegia (age of onset of symptoms, presence of paroxysmal and non-paroxysmal features, seizures, and dysautonomia), cardiac comorbidities, medication use at the time of ECG recordings, and family history of cardiac disease and sudden cardiac or unexplained death were collected by collaborating physicians, and subsequently analysed.

Patients data from previously published studies were analysed by whole-exome or whole-genome sequencing according to published, or local, protocols (Supplementary material) (Heinzen *et al.*, 2012; Rosewich *et al.*, 2014). Direct Sanger sequencing of *ATP1A3* was undertaken in cases where mutation status was unknown (Supplementary material). *De novo* mutation status was evaluated by Sanger sequencing where parental DNA was available; where unavailable, pathogenicity was declared if the mutation was previously reported as *de novo* in another patient. Cases where no mutation in *ATP1A3* was identified were included if they met the clinical diagnostic criteria for alternating hemiplegia.

Original ECG records were scanned, collected and reviewed centrally. For one UK patient, only serial historical ECGs were available. Five patients had serial 12-lead ECGs available (four had two ECGs, and one patient had three). All 12-lead ECGs were recorded at a paper speed of 25 mm/s and amplitude of 10 mm/mV, and evaluated independently by three cardiologists with expertise in cardiac electrophysiological disease, sudden cardiac death and inherited cardiac disease (P.D.L., E.R.B., J.P.K.). Abnormal repolarization was defined by the presence of abnormal T wave morphology (flattened or biphasic T waves; bifid or notched T waves) or T wave inversion in two or more of the following leads: I, aVL and V4–V6 (lateral repolarization abnormalities); II, III and aVF (inferior repolarization abnormalities); and V1–V3 in patients aged ≥14 years (anterior repolarization abnormalities); repolarization abnormalities of this type are seen in 2% of healthy adults (Rautaharju *et al.*, 2009). The corrected QT interval was calculated from lead II using Bazett’s formula (Bazett 1920); its normal range is 360–460 ms (Priori *et al.*, 2013); J-point elevation and early repolarization were defined as previously described (Junttila *et al.*, 2012), and are seen in 1–5% of healthy individuals (Klatsky *et al.*, 2003). Right bundle branch block (complete and incomplete) and intraventricular conduction delays (IVCDs) were defined according to established criteria (Surawicz *et al.*, 2009). Isolated IVCD was considered normal in the absence of additional ECG abnormalities, as it is seen in up to 5% of the normal population (Chiu *et al.*, 2008; Bussink *et al.*, 2013). Isolated right bundle branch block is seen in 2–4% of healthy individuals (Bussink *et al.*, 2013). Four patients (Patients 1, 37, 41 and 50; Tables 1 and 3) also had EEG-videotelemetry recording (25–98 h), which included single-lead ECG (modified V1). Data from the previously-reported patient (Patient 1) were re-evaluated, given the novel findings from this study (Novy *et al.*, 2014).

**Table 1:** Clinical neurological features and mutation status in patient cohort

Patient/gender	Age of onset (months)	Paroxysmal features							Non-paroxysmal features							
		c.DNA change	Amino acid change	Plegic attacks	Dystonia	Seizures	Abnormal oculomotor	Autonomic	Pyramidal	Ataxia/dysarthria	Dystonia	Muscle tone	Complex movement disorder	Other non-paroxysmal features	Developmental and/or intellectual delay	Behavioural disturbance
1 F	0	c.410C>T	p.S137F	+	+	+	+^a^	+ Asystolic periods	+	+/+	+	Hypertonia	+	Migraine	+	−
2 M	1	c.410C>T	p.S137F	+	+	+ and status	+^a^	+ Dyspnoea, altered HR and apnoeic episodes	+	+/−	−	Hypotonia	−	−	+	−
3 M	0	c.821T>A	p.I274N	+	+	−	+^a^	+	−	−/−	+	Normal	−	−	+	−
4 M	29	c.829G>A	p.E277K	+	+	+	+	−	−	−/+	+	Hypotonia	−	−	−	−
5 F	18	c.1010T>G	p.L337R	+^a^	−	+	−	−	+	+/+	+	Hypertonia	+	Pre-syncopal episodes and palpitations, migraine with aura	−	−
6 M	3	c.2263G>A	p.G755S	+	+	+	+^a^	+	−	+/+	+	Hypertonia	+	Bulbar Symptoms	+	−
7 M	9	c.2314A>C	p.S772R	+^a^	+	+ and status	−	−	+	+/+	+	Hypertonia	+	Opsoclonus, migraine	+	−
8 M	3	c.2401G>A	p.D801N	+^a^	+	−	+	+ Sweating	+	+/NK	+	Hypertonia	−	−	+	−
9 F	0	c.2401G>A	p.D801N	+	+	−	+^a^	+ Dyspnoea	+	+/+	+	Hypertonia	−	Complex oculomotor disturbance with opsoclonus and migraine	+	−
10 F	1	c.2401G>A	p.D801N	+	+^a^	−	+	+	+	−/−	+	Hypertonia	+	−	+	−
11 M	17	c.2401G>A	p.D801N	+^a^	+^a^	−	+^a^	+	−	−/+	+	Normal	−	Deviated nasal septum.	+	+
12M	1	c.2401G>A	p.D801N	+	+^a^	+	+	−	−	+	+	Hypertonia	+	Bulbar symptoms	+	+
13 F	12	c.2401G>A	p.D801N	+	+	+^a^	+	+	−	+/very mild ataxia	+	Normal	+	Bulbar symptoms	+/−	+
14 M	2	c.2401G>A	p.D801N	+	+	+	+^a^	+	?	+	+	Normal	+	Bulbar Symptoms	+/−	−
15 M	4	c.2401G>A	p.D801N	+	+	−	+	−	−	−/+	+	Hypertonia	+	−	+	+
16 M	2	c.2401G>A	p.D801N	+	+	−	+^a^	−	−	−/−	−	Hypotonia	−	−	+	−
17 F	3	c.2401G>A	p.D801N	+^a^	+	+^a^	+	−	−	−/−	+	Hypotonia	−	−	+	−
18 M	0	c.2401G>A	p.D801N	+^a^	+	+	+^a^	−	+	+/+	+	Normal	+	Migraine	+	+
19 F	0	c.2401G>A	p.D801N	+^a^	+	+^a^	+	+	+	+/+	+	Hypotonia	+	Tremor	+	+
20 M	5	c.2401G>A	p.D801N	+^a^	+	+	+	+	+	+/+	+	Hypotonia	+	Migraine	+	+
21 F	2	c.2401G>A	p.D801N	+^a^	+	+	+^a^	−	−	+/+	+	Hypotonia	+	Non-migrainous headache	+	+
22 F	4	c.2401G>A	p.D801N	+	+^a^	+	+	+	+	+/+	−	Hypertonia	−	−	+	+
23 F	4	c.2401G>A	p.D801N	+	+^a^	+	+	−	+	+/+	−	Hypotonia	−	Non-migrainous headache	+	−
24 F	7	c.2401G>A	p.D801N	+^a^	+	+	+	+	+	+/+	+	Hypotonia	−	−	+	−
25 F	1	c.2401G>A	p.D801N	+	+	+	+^a^	+	+	−/+	−	Hypertonia	−	Non-migrainous headache	+	−
26 F	1	c.2401G>A	p.D801N	+ (U)	+	+ and status	+^a^	−	+	+/+	+	Hypotonia	+	Migraine	+	+
27 F	5	c.2411C>T	p.T804I	+^a^	+	−	+	−	−	+/+	−	Hypotonia	−	−	+	−
28 M	13	c.2417T>G	p.M806R	+^a^	+	+	+^a^	−	−	−/NK	+	Hypotonia	−	Non-migrainous headache	+	−
29 F	1	c.2431T>C	p.S811P	+^a^	−	+	+^a^	+	+	+/+	+	Hypertonia	−	Regional pain syndrome and skin colour change; migraine	+	−
30 F	0	c.2443G>A	p.E815K	+	+	+^a^	+	+	−	−/NA	−	Hypotonia	−	−	+	−
31 M	4	c.2443G>A	p.E815K	+	+	+ and status	+	+	+	+	+	Hypotonia	+	Intermittent pain and altered skin temperature of limbs	+	−
32 M	1.5	c.2443G>A	p.E815K	+	+^a^	+	+^a^	+	+	+/+	+	Hypotonia	+	−	+	−
33 M	1	c.2443G>A	p.E815K	+	+	+	+^a^	+	+	+/+	+	Hypotonia	+	−	+	+
34 F	1	c.2443G>A	p.E815K	+ (U)	+	+	+^a^	+	+	+/+	+	Hypotonia	−	−	+	+
35 F	1	c.2443G>A	p.E815K	+	+	+	+^a^	+	−	NK/−	−	Hypotonia	−	−	+	−
36 M	0	c.2443G>A	p.E815K	+^a^	+	+	+^a^	+	−	+/+	+	Normal	−	−	+	+
37 F	0	c.2443G>A	p.E815K	+	+	+ and status	+^a^	NK	−	−/NK	+	Hypotonia	−	Complex generalised dystonia, orofacial, limb, eye movements	+	
38 F	6	c.2443G>A	p.E815K	+	+	+ and status	+^a^	+	−	+/NA	+	Normal	+	−	+	+
39 M	0	c.2755_2757delGTC	p.V919del	+	+^a^	−	+^a^	+	+	−/+	+	Hypotonia	+	−	+	+
40M	1	c.2767G>T	p.D923Y	+	+^a^	+	+	+ intermittent pallor	−	+/+	+	Hypertonia	+	Bulbar and respiratory disturbance	+	−
41 M	4	c.2781C>T	p.C927W	+	+^a^	+ and status	+	+	+	+/+	+	Hypertonia	−	Migraine	+	+
42 F	1	c.2839G>A	p.G947R	+	+	+^a^	+	+	−	+/+	+	Hypotonia	−	−	+	−
43 F	1	c.2839G>A	p.G947R	+ (U)	+	+ and status	+^a^	+	+	+/+	+	Hypotonia	+	−	+	−
44 F	3	c.2839G>A	p.G947R	+	+	−	+^a^	−	+	−/−	+	Hypertonia	+	Migraine	+	−
45M	2	c.2839G>A	p.G947R	+	+	−	+^a^	−	−	+/+	+	Normal	−	−	+/−	−
46 M	0	c.2839G>A	p.G947R	+	−	+	+	−	−	+/+	−	Normal	−	−	+	NK
47M	0	c.2839G>A	p.G947R	+	+	+	−	−	−	−/+	+	Hypotonia	+	Non-migrainous headache	+	−
48 M	0	No mutation		+^a^	+^a^	−	+^a^	+	−	−/−	+	Hypotonia	+	Headache - unspecified	+	+
49 F	4	No mutation		+^a^	NK	+	−	+ Altered heart rate, and body temperature	−	+/NK	NK	Normal	−	Migraine	+	+
50F	5	No mutation		+^a^	−	−	+^a^	−	−	+/+	+	Hypotonia	−	−	+	−
51 M	8	No mutation		+^a^	+	+	−	−	−	−/+	−	Normal	+	−	+	+
52 F	7	No mutation		+^a^	+^a^	+	+^a^	+	+	−/−	+	Normal	−	−	+	+

+^a^ = symptom at onset; + denotes symptoms present; − indicates absence of symptom; HR =; NK = not known; NA = not applicable; U = unilateral.

### Statistical analyses

Age-related differences in ECG abnormalities were calculated using Fisher’s exact test, and differences in mean corrected QT interval (QTc) between groups using the unpaired *t*-test. All analyses were performed using the Statistical Package for Social Sciences Software programme (IBM SPSS Statistics, Version 22.0., IBM Corp). A Bonferroni correction was applied where appropriate.

## Results

### Demographics

We analysed ECG data of 52 patients with alternating hemiplegia, from nine countries: Spain (*n = *14); UK (*n = *13); Italy (*n = *7); Germany (*n = *7); USA (*n = *6); Canada (*n = *2); Australia (*n = *1); France (*n = *1); and Portugal (*n = *1). Twenty patients were aged 16 years or over; 32 patients were under 16; 26 were female, 26 male (see Table 1). There was no significant difference in mean age between people with alternating hemiplegia (173.8 months) and the disease controls (176.3 months) (paired *t*-test, two-tailed, *P = *0.166).

### Molecular genetics

Forty-seven patients had a confirmed missense mutation in *ATP1A3* identified either through previous whole-exome sequencing (Heinzen *et al.*, 2012; Rosewich *et al.*, 2014), or sequencing in this study (Table 2). The most frequent mutation observed was c.2401G > A; p.D801N (*n = *19; 36.5%) followed by c.2443G > A; p.E815K (*n = *9; 17.3%), in keeping with previous reports (Heinzen *et al.*, 2012; E. Panagiotakaki, personal communication). Mutations c.2443G > A, p.S772R; c.2411C > T, T804I; c.1010T > G, L337R; and c.2781C > T, p.C927W have recently been reported (E. Panagiotakaki, personal communication). One patient (Patient 37) had a 3-bp deletion. No mutation in *ATP1A3* was found in five patients after targeted next-generation gene sequencing, whole-exome or genome sequencing.

**Table 2:** Summary of mutation status in ECG study cohort

Nucleotide change	Amino acid change	Exon	Number of probands (%)
c.410C>T	p.S137F	5	2 (3.8)
c.821T>A	p.I274N	8	1 (1.9)
c.829G>A	p.E277K	8	1 (1.9)
c.1010T>G	p.L337R	9	1 (1.9)
c.2263G>A	p.G755S	17	1 (1.9)
c.2314A>C	p.S772R	17	1 (1.9)
c.2401G>A	p.D801N	17	19 (36.5)
c.2411C>T	p.T804I	17	1 (1.9)
c.2417T>G	p.M806R	17	1 (1.9)
c.2431T>C	p.S811P	18	1 (1.9)
c.2443G>A	p.E815K	18	9 (17.3)
c.2755_2757delGTC	p.V919del	20	1 (1.9)
c.2767G>T	p.D923Y	20	1 (1.9)
c.2781C>T	p.C927W	20	1 (1.9)
c.2839G>A	p.G947R	21	6 (11.5)
No mutation			5 (9.6)
Total			52

**Table 3:** Mutation status and ECG abnormalities in the study cohort

Patient	Age at ECG	Mutation status		Medications at time of ECG	ECG findings							
					Repolarization abnormality				IVCD	Incomplete RBBB	J wave changes	Other
					Anterior	Lateral	Inferior	Widespread				
1	21 years	c.410C>T	p.S137F	Flunarizine, pizotifen, carbamazepine	−	−	−	−	−	−	−	−
	23 years (VTM)			Flunarizine, pizotifen, carbamazepine	NA	NA	NA	NA	NA	NA	NA	Modified V1 on VTM normal
	23 years (ILR)			Flunarizine, pizotifen, carbamazepine	NA	NA	NA	NA	NA	NA	NA	Asystolic periods >3 s on ILR
2	7 years	c.410C>T	p.S137F	Flunarizine, topiramate, melatonin, midazolam	−	−	−	−	−	−	−	−
3	12 years	c.821T>A	p.I274N	Flunarizine, risperidone	−	−	−	−	+*	−	−	TWI V1-V2*
4	2 years, 5 months	c.829G>A	p.E277K	Prednisolone, IVIg 1 day before ECG, trihexylphenidyl	−	−	−	−	−	−	−	−
5	27 years	c.1010T>G	p.L337R	Acetazolamide, pregabalin, lamotrigine	−	−	−	+	+	−	−	−
	33 years			Acetazolamide, pregabalin, lamotrigine	−	−	−	+	+	−	−	−
6	10 years	c.2263G>A	p.G755S	Topiramate	−	−	−	−	+*	−	−	−
7	18 years	c.2314A>C	p.S772R	Flunarizine, topiramate, sumatriptan, midazolam	+	−	+	−	−	+	−	RAD
	19 years			Flunarizine, topiramate, midazolam, pizotifen	−	−	+	−	+	−	−	−
8	18 years	c.2401G>A	p.D801N	−	+	−	−	−	−	+	−	−
9	25 years	c.2401G>A	p.D801N	Sodium valproate, clobazam, quetiapine, lorazepam, sertraline	+	−	+	−	+	−	ERP leads I and aVL	TWI V2, flat T wave V3
	25 years			Sodium valproate, clobazam, quetiapine, lorazepam, sertraline	+	−	+	−	+	−	ERP leads I and aVL	TWI V1-V3
10	14 years, 10 months	c.2401G>A	p.D801N	Flunarizine	−	−	−	+	−	−	−	−
11	9 years	c.2401G>A	p.D801N	−	−	−	−	−	+ *	−	−	TWI V1-V3*
12	30 years	c.2401G>A	p.D801N	−	−	+	−	−	−	−	ERP inferior leads	Indeterminate BBB, RAD
13	15 years	c.2401G>A	p.D801N	Flunarizine, risperidone	+	−	+	−	−	−	−	RAD
14	10 years	c.2401G>A	p.D801N	−	−	+	−	−	−	−	Subtle ERP inferior leads	−
15	3 years, 11 months	c.2401G>A	p.D801N	Flunarizine, clonazepam, topiramate	−	−	−	−	+ *	−	−	−
	9 years, 3 months			Lorazepam, chlorzoxazone	−	−	+	−	−	+	−	−
16	3 years	c.2401G>A	p.D801N	Flunarizine	−	−	−	−	−	−	−	−
17	1 year, 10 months	c.2401G>A	p.D801N	Flunarizine, calcium supplements, omega 3, potassium phosphate	−	−	−	−	−	−	−	−
18	7 years	c.2401G>A	p.D801N	Flunarizine, lamotrigine, melatonin	−	−	−	−	−	+	Notching of terminal portion of QRS V1	−
19	4 years	c.2401G>A	p.D801N	Flunarizine, topiramate, clonazepam, esomeprazole, ranitidine	−	−	−	−	−	−	−	−
20	18 years	c.2401G>A	p.D801N	Flunarizine, levetiracetam, topiramate, olanzapine	−	−	−	+	−	+	−	Frequent monomorphic VEs
21	21 years	c.2401G>A	p.D801N	Topiramate, clonazepam, cinarizine	−	−	−	+	+	−	Dynamic 1 mm J-point elevation V1	−
22	8 years	c.2401G>A	p.D801N	Flunarizine, ketogenic diet, carnitines, vitamins	−	−	−	−	−	−	−	TWI V1-V3*
23	31 years	c.2401G>A	p.D801N	Carbamazepine, topiramate	−	−	+	−	−	−	−	−
24	27 years	c.2401G>A	p.D801N	Flunarizine, topiramate, clobazam	+	−	+	−	−	+	−	LAD
25	28 years	c.2401G>A	p.D801N	Flunarizine, sodium valproate, clobazam	−	+	+	−	+	−	−	−
26	14 years, 5 months	c.2401G>A	p.D801N	Flunarizine, sodium valproate, trihexiphenidyl	+	−	+	−	−	+	−	−
27	11 years, 5 months	c.2411C>T	p.T804I	Flunarizine, ketogenic diet, vitamins	−	−	+	−	+	−	−	−
28	2 years, 4 months	c.2417T>G	p.M806R	Flunarizine	−	−	−	−	−	−	−	−
29	26 years	c.2431T>C	p.S811P	Flunarizine, topiramate, phenytoin, midazolam	−	−	−	+	+	−	−	RAD
30	1 year, 2 months	c.2443G>A	p.E815K	−	−	−	−	−	+ *	−	−	TWI V1-V3*
31	25 years	c.2443G>A	p.E815K	Flunarizine, zonisamide, sodium valproate, levetiracetam, oxcarbezepine, lacosamide, clobazam, domperidone, esomeprazole, vitamin D, colestyramine, L-carnitine	−	−	−	−	+ *	−	−	−
32	8 years	c.2443G>A	p.E815K	Clobazam, lamotrigine	−	−	−	−	−	−	−	−
33	8 years	c.2443G>A	p.E815K	−	−	−	−	−	−	+ *	−	TWI V1 V3*
34	13 years, 9 months	c.2443G>A	p.E815K	Flunarizine, lamotrigine, clonazepam, pregabalin, omeprazole	+	−	+	−	+	−	−	RAD
35	3 years, 1 months	c.2443G>A	p.E815K	Flunarizine, levetiracetam, vitamins, bicarbonate	+	−	+	−	+	−	−	−
36	5 years, 2 months	c.2443G>A	p.E815K	Flunarizine, sodium valproate, clobazam, trihexylphenidyl	−	−	−	−	−	−	−	−
37	24 years	c.2443G>A	p.E815K	Flunarizine, phenytoin, pregabalin, clobazam, levetiracetam, ranitidine, hyoscine, domperidone	+	−	+	−	−	+	−	−
	24 years (VTM)			Flunarizine, phenytoin, pregabalin, clobazam, levetiracetam, ranitidine, hyoscine, domperidone	NA	NA	NA	NA	NA	NA	NA	Modified V1 on VTM normal
38	5 years, 6 months	c.2443G>A	p.E815K	Flunarizine	−	+	+	−	−	−	−	−
39	0	c.2755_2757 delGTC	p.V919del	−	−	−	−	−	−	−	−	TWI V1-V3*
	2 days			−	−	−	−	−	−	−	−	TWI V1-V3*
	8 months			−	−	−	−	−	−	−	−	TWI V1-V3*
	20 years, 8 months (VTM)			Flunarizine, acetazolamide, tryptophan	NA	NA	NA	NA	NA	NA	NA	V1 on VTM normal
40	20 years	c.2767G>T	p.D923Y	Sodium valproate, risperidone, memantine	−	−	+	−	−	−	Inferior and lateral ERP	−
41	38 years	c.2781C>T	p.C927W	Lamotrigine, clonazepam, risperidone, omeprazole, clomipramine clorhydrate	−	−	−	−	+ *	−	−	−
42	15 years, 10 months	c.2839G>A	p.G947R	Flunarizine, clonazepam, vitamins, L-Dopa/carbidopa	−	+	+	−	−	−	−	−
43	7 years, 11 months	c.2839G>A	p.G947R	Flunarizine, clonazepam, carbamazepine	−	−	−	−	−	−	−	−
44	35 years	c.2839G>A	p.G947R	Baclofen	−	−	−	−	+ *	−	−	−
	35 years (VTM)			Baclofen	NA	NA	NA	NA	NA	NA	Dynamic J-point elevation (modified V1)	−
45	3 years, 10 months	c.2839G>A	p.G947R	−	−	−	−	−	−	−	−	−
46	35 years	c.2839G>A	p.G947R	Carbamazepine	−	+	−	−	−	−	−	−
47	23 years	c.2839G>A	p.G947R	Carnitines	+	−	−	−	+	−	−	−
48	4 years, 10 months	No mutation		−	−	−	−	−	−	−	TWI V1-V2, biphasic T waves V3*
49	30 years	No mutation	Flunarizine, pizotifen, diazepam, baclofen, zonisamide	−	−	−	−	−	−	−	−
50	1 years, 6 months	No mutation	None	−	−	−	−	+ *	−	−	−
51	10 years, 5 months	No mutation	Flunarizine, tri-hexylphenidyl, clobazam, melatonin	−	−	−	−	+ *	−	−	−
52	4 years	No mutation	Flunarizine, amitryptilline, clonidine	−	−	−	−	−	+ *	−	−

*Normal for age; + denotes presence of ECG abnormality; - indicates absence of abnormality; (R)BBB = right bundle branch block; ERP = early repolarization; ILR = implantable cardiac loop recorder device; IVCD = intraventricular conduction delay; IVIg = intravenous immunoglobulins; LAD = left axis deviation; NA = not applicable; RAD = right axis deviation; TWI = T wave inversion; VE = ventricular extrasystole; VTM = EEG-videotelemetry monitoring.

### Clinical autonomic and cardiac features in patients with alternating hemiplegia

Autonomic features were reported in 32 patients (62%) during plegic episodes (Table 1). Altered heart rate and apnoeic episodes were reported by the carers of Patient 2, and tachycardia and altered body temperature was documented in the medical records of Patient 49. Three patients reported at least one episode of palpitation in isolation, without syncope. One subject (Patient 1) started experiencing episodes of loss of consciousness with respiratory arrest at the age of 21 years (Novy *et al.*, 2014). Her routine 12-lead ECG recording was normal. She underwent implantation of a cardiac loop recorder, which documented three episodes of asystole longer than 3 s over a period of 4 months: a cardiac pacemaker was implanted. She had had EEG-videotelemetry prior to pacemaker implantation. The single-lead ECG that was part of the telemetry showed sinus rhythm throughout, with no arrhythmias or changes in QRS, J-point or T wave morphology.

### Electrocardiographic features in disease controls

Repolarization abnormalities were seen in 5/52 disease controls, isolated to inferior leads in one, inferolateral in one and widespread in three. Isolated anterior, lateral or infero-anterior changes were not seen. IVCD was noted in 9/52 (17.3%), and incomplete right bundle branch block in separate 6/52 (11.5%) disease controls. Early repolarization was seen in 3/52 (5.8%), whereas none had J-wave changes, or IVCD/right bundle branch block in combination with pathological ECG findings. Data from these disease controls are provided in Supplementary Table 1.

### Electrocardiographic features in patients with alternating hemiplegia

Table 3 shows the ECG features of the study population. Overall, ECG records were abnormal in 28 cases, with the resting 12-lead ECG abnormal in 26 patients (50%). Some changes were subtle. Seven of 52 (13.5%) disease control ECGs were deemed abnormal using the same criteria, significantly fewer than the alternating hemiplegia group (Fisher’s exact test, two-tailed, *P = *0.0001).

**Figure 2:**
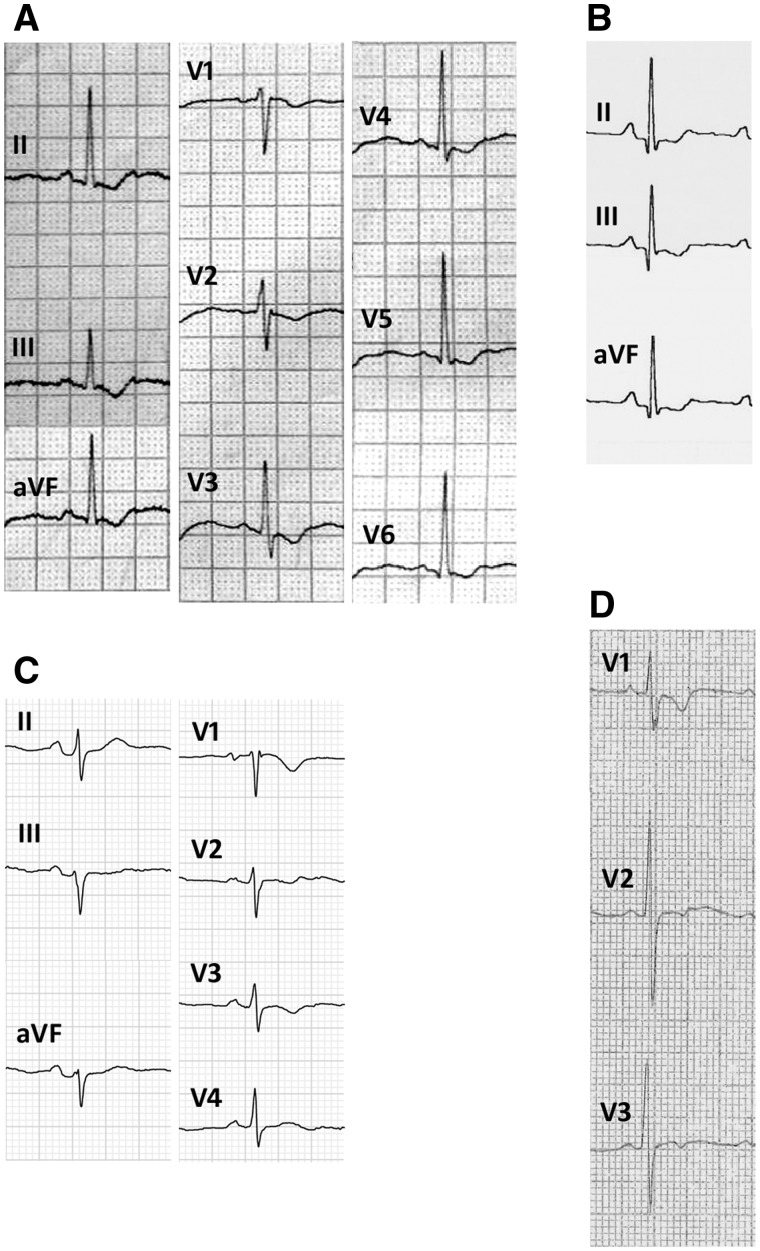
**Repolarization abnormalities.** Examples of ECG recordings showing widespread repolarization abnormalities in Patient 5 (**A**), isolated inferior repolarization abnormalities in Patient 23 (**B**), inferior and anterior repolarization abnormalities in Patient 24 (**C**), and isolated anterior repolarization abnormalities in Patient 47 (**D**).

**Figure 3:**
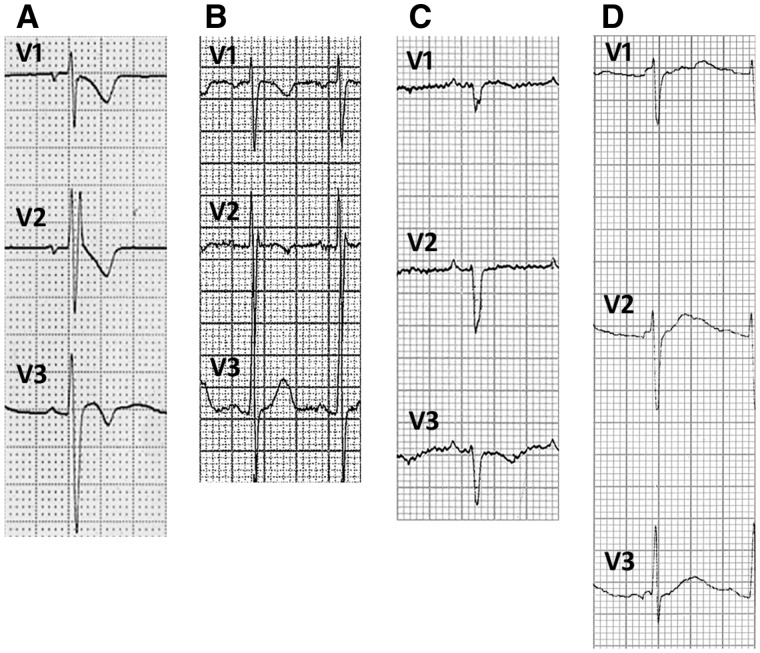
**Intraventricular conduction delay.** Examples of ECG recordings showing incomplete right bundle branch block (RBBB) and anterior repolarization abnormalities in Patient 8 (**A**), incomplete right bundle branch block in Patient 52 (**B**), IVCD and anterior repolarization abnormalities in Patient 29 (inferior and lateral repolarization abnormalities not shown) (**C**), and minor IVCD in Patient 31 (**D**).

**Figure 4:**
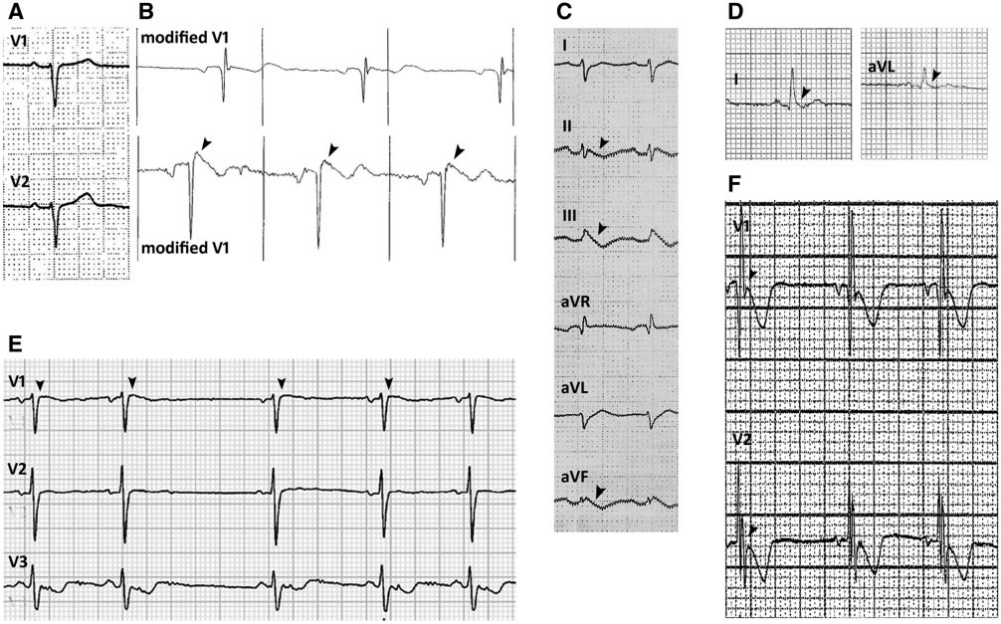
**J-point changes.** Leads V1 and V2 of the normal baseline 12-lead ECG in Patient 44 (**A**). The same patient had a single lead (modified V1) ECG recording during video-telemetry, showing dynamic features of Brugada syndrome. While the top tracing is normal, the bottom tracing, recorded at a different time during the same recording, shows mild prolongation of QRS and J-point elevation (arrowheads) (**B**). Marked early repolarization in inferior leads (arrowheads) in Patient 12 (**C**). Lateral early repolarization (arrowheads) in Patient 9 (inferior and anterior repolarization abnormalities not shown) (**D**). Dynamic J-point elevation in V1 (arrowheads) in Patient 21 (**E**). Notching of the terminal portion of QRS in V1 in Patient 18 (**F**).

**Figure 5:**
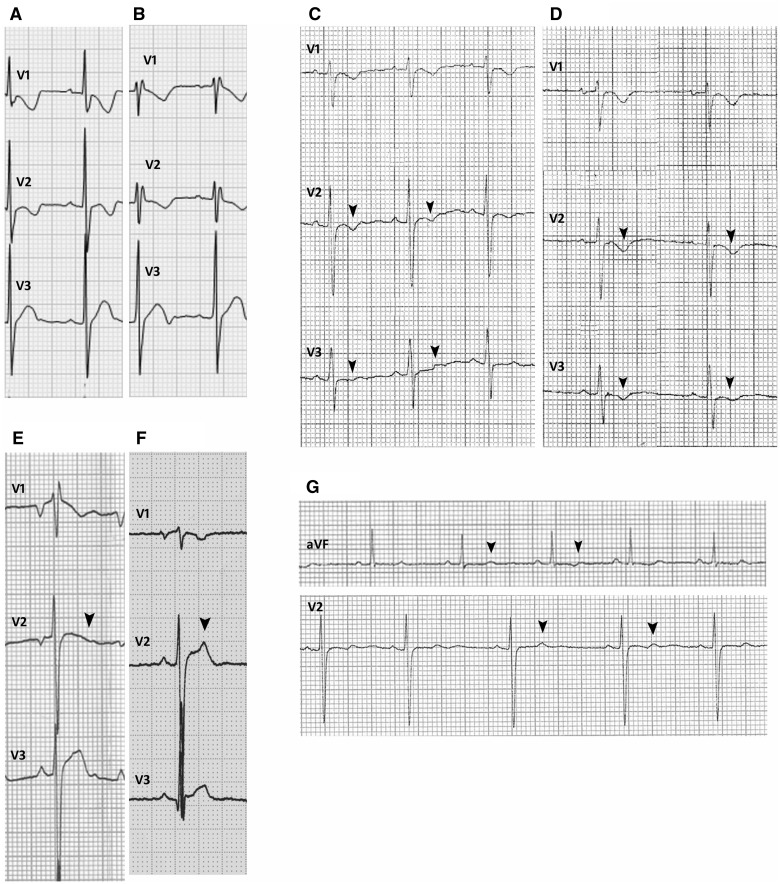
**Age-related changes and dynamic changes.** The baseline ECG performed in Patient 15 at the age of 3 years shows minor IVCD (**A**). The ECG performed at the age of 9 years in the same subject shows incomplete right bundle branch block [inferior repolarization abnormalities not shown (**B**)]. Dynamic anterior repolarization abnormalities in Patient 9: biphasic T-waves (arrowheads) in baseline ECG (**C**) and inverted T-waves (arrowheads) in the ECG recorded a week later than the baseline ECG (**D**). The baseline ECG performed at the age of 18 years in Patient 7 shows incomplete right bundle branch block, anterior repolarization abnormalities and right axis deviation [inferior repolarization abnormalities not shown (**E**)]. The ECG performed at the age of 19 years in the same case shows IVCD and no anterior repolarization abnormalities [arrowheads (**F**)]. Inferior and lateral dynamic repolarization abnormalities with subtle beat-to-beat variation (arrowheads) in T-waves in Patient 10 (**G**).

Repolarization abnormalities were present in 25 patients (48.1%). The prevalence of repolarization abnormalities in the alternating hemiplegia cases was significantly higher than in the disease control group (25/52 versus 5/52 respectively; Fisher’s exact test, two-tailed, *P < *0.0001). Co-existing ECG abnormalities included IVCD (*n = *10, 19.2%), incomplete right bundle branch block (*n = *8, 15.4%); left axis deviation (*n = *1, 1.9%), right axis deviation (*n = *5, 9.6%), lateral early repolarization (*n = *1, 1.9%) and inferior early repolarization (*n = *3, 5.8%) (distinct from ‘repolarization abnormality’). Data from a single-lead ECG during EEG-videotelemetry were available for four patients. No supraventricular or ventricular arrhythmias were detected, even during plegic episodes. However, one patient with a normal resting 12-lead ECG had dynamic J-point elevation in modified lead V1 on EEG-videotelemetry recording (see below). Asystole was detected in one patient by an implantable loop recorder, as previously reported. Figures 2–5 show illustrative segments from abnormal ECGs.

The use of flunarizine or not at the time of ECG was not associated with ECG abnormalities (Fisher’s exact test, two-tailed, *P = *1.0). The use or not of any antiepileptic drug was not associated with ECG abnormalities (Fisher’s exact test, two-tailed, *P = *0.094).

### Repolarization abnormalities

Repolarization abnormalities consisted of T wave inversion, and/or abnormal T wave morphology. The average QTc interval in all alternating hemiplegia patients was 394 ms (range 350–440 ms). In the 52 disease controls, the mean QTc was 418ms (range 380–460 ms, within the normal range). Overall, the QTc interval was significantly shorter in the alternating hemiplegia cases compared with the disease control group (unpaired *t*-test, two-tailed, *P < *0.0001). Four patients (7.7%) had isolated inferior repolarization abnormalities, two (3.8%) had isolated anterior repolarization abnormalities, three (5.8%) had infero-lateral repolarization abnormalities, eight (15.4%) had infero-anterior repolarization abnormalities and five (9.6%) had widespread repolarization abnormalities in the anterior, inferior and lateral leads (Table 3 and Fig. 2).

### Intraventricular conduction delay

IVCD (*n = *20) or incomplete right bundle branch block (*n = *10) were present in 28 individuals (53.8%), including 17 with concomitant repolarization abnormalities. Of the 26 patients with a normal resting 12-lead ECG, 10 (38.5%) had IVCD in lead V1, and two (3·8%) had incomplete right bundle branch block (Table 3 and Fig. 3). The prevalence of IVCD or right bundle branch block was significantly more common in alternating hemiplegia than in the disease control cohort (28/52 versus 15/52; Fisher’s exact test, two-tailed, *P = *0.0164).

### J wave changes

One patient (Patient 44) showed transient asymptomatic cove-shaped ST segment elevation (J-point elevation), characteristic of Brugada syndrome, on single-lead ECG recording during EEG-videotelemetry (Fig. 4A and B). One individual (Patient 21) had intermittent, dynamic 1 mm J-point elevation in lead V1 (see below; Fig. 4E); a further individual (Patient 18) had prominent notching of the terminal portion of the QRS complex without J-point elevation (Fig. 4F) and four patients (Patients 9, 12, 14 and 40) had early repolarization changes associated with repolarization abnormalities (Fig. 4C and D).

### Changes with age and related to specific mutation

One individual (Patient 15) had a normal ECG with IVCD at the age of 3 years; at age 9 years, incomplete right bundle branch block and abnormal repolarization inferiorly were noted (Fig. 5A and B). Dynamic changes were also seen in Patient 7 (Fig. 5E and F). Overall, the prevalence of ECG abnormalities was significantly greater in individuals aged ≥ 16 years than in those < 16 years (*P = *0·0095). Nineteen patients harboured the p.D801N mutation: all eight patients (42·1%) ≥ 16 years, but only 6/11 patients (18·8%) < 16 years, had abnormal ECGs (*P = *0·045).

The prevalence of any ECG abnormalities, and of repolarization abnormalities, remained significantly higher in the alternating hemiplegia cohort than in the disease control cohort if only the 47 cases with alternating hemiplegia with *ATP1A3* mutation were considered (*P < *0.0001 for both comparisons). The QTc interval also remained significantly shorter when comparing only the 47 alternating hemiplegia cases with *ATP1A3* mutation against all 52 disease controls (unpaired *t*-test, *P < *0.0001).

### Dynamic ECG changes

Three of five patients in whom serial 12-lead ECGs were available had dynamic electrocardiographic changes that varied from one ECG to another. Patient 9 had dynamic T wave inversion in leads V1–V3 (Fig. 5C and D). Six individuals (11.5%) had dynamic beat-to-beat ECG changes: five had dynamic changes in the T wave morphology (Fig. 5G), and one individual had intermittent 1 mm J-point elevation in lead V1 (Fig. 4E).

## Discussion

Alternating hemiplegia is a rare neurological disorder with significant phenotypic diversity (Panagiotakaki *et al.*, 2010). Known outcomes range from life into adulthood, with comparatively little disability, to premature mortality from sudden death. The broad range of presentations has typically been ascribed to neurological abnormalities, including epilepsy-related sudden death (SUDEP). Discovery of the underlying cause of most cases, *de novo* mutation in *ATP1A3*, is accelerating understanding of alternating hemiplegia (Heinzen *et al.*, 2014). *ATP1A3* expression extends beyond the brain, and includes the heart (Aye *et al.*, 2010). In keeping with this expression pattern and both paroxysmal and interictal neurological dysfunction in *ATP1A3*-related disease (Heinzen *et al.*, 2014), we show common and dynamic abnormalities of cardiac physiology in alternating hemiplegia, as manifest in electrocardiographic data. Our findings have implications for the more complete understanding and management of alternating hemiplegia, and other cardiocerebral disorders, which include many epilepsies. The data also indicate the need for caution with drugs used for other symptoms or problems in people with alternating hemiplegia, as is the case, for example, with Brugada syndrome.

Overall, we show some type of ECG abnormality in just over half the cases (28/52). These abnormalities fall into three main categories: abnormal repolarization, with or without IVCD or incomplete right bundle branch block; J-wave or J-point changes; and the previously-reported single case of asystole. Repolarization abnormalities were present in 25 patients (48.1%), whereas they are seen in only 2% of healthy adults (Rautaharju *et al.*, 2009). While isolated IVCD and incomplete right bundle branch block changes can be normal findings, the prevalence in our cohort (21.2%) is much higher than published normal data [2.3% in females; 4.7% in males (Bussink *et al.*, 2013)], particularly in children [∼1% (Chiu *et al.*, 2008)], and much higher than the prevalence in disease controls with epilepsy. In addition, corrected QT intervals were significantly shorter in the alternating hemiplegia cohort compared to epilepsy disease controls. Short QT syndrome is a relatively recently-described cardiac channelopathy associated with a high risk of ventricular arrhythmia and sudden death (Priori *et al.*, 2013), and mutations in *KCNJ2* have recently been reported in patients with short QT syndrome and an autism–epilepsy phenotype (Ambrosini *et al.*, 2014). In contrast, QT prolongation (rather than shortening) has been reported in individuals with epilepsy (Surges *et al.*, 2010), suggesting that if alternating hemiplegia has an effect on the QT interval, it is the opposite of that seen in people with epilepsy. These findings are intriguing, but will require more data, possibly including longitudinal data, to interpret.

Several of the characteristics of the changes observed are typical of inherited cardiac channelopathies: the waveforms themselves, emergence with age, and beat-to-beat or ECG-to-ECG variation. In one case, a transient waveform was typical of that seen in Brugada syndrome, an inherited cardiac electrophysiological disorder most commonly associated with loss-of-function mutations in the cardiac sodium channel gene *SCN5A* (in 20–30% of cases; Priori *et al.*, 2013). Dynamic ECG changes are known to occur in many genetic cardiac channelopathies. A study of 89 patients with Brugada syndrome who underwent implantable cardiovertor defibrillator insertion and had serial ECG recordings revealed that only 24% of all ECGs per patient showed the diagnostic coved-type ST-segment elevation, 25% showed non-diagnostic ST-segment changes, and 51% were normal (Richter *et al.*, 2009). Studies of serial ECGs in patients with long QT syndrome revealed considerable variability in QTc interval duration, with some measurements falling within the normal range (Goldenberg *et al.*, 2006; Lee *et al.*, 2013). The observed transience of the abnormalities recorded in our cohort suggests our findings, based largely on standard brief interictal ECG records, may underestimate the true prevalence of ECG abnormalities in alternating hemiplegia, and point to the need for systematic studies with longer ECG recordings.

ECG abnormalities were more common in patients 16 years or older compared with those under 16. The p.D801N, p.E815K and p.G947R mutations are the most common mutations reported; p.E815K is generally associated with the most severe course of disease (Sasaki *et al.*, 2014). In our cohort of patients, the most frequent mutation identified was pD801N (36.5%), followed by c.2443G > A; p.E815K (17.3%), and c.2839G > A; p.G947K (11.5%), consistent with published data. Overall, 73.7% of those harbouring D801N mutations had abnormal ECG recordings; 57% of those with abnormalities were aged over 16 (Table 3). Age-related penetrance of cardiac conduction abnormalities has been described in other cardiac channelopathies. In *SCN5A* mutation-positive patients with Brugada syndrome, intraventricular conduction changes were found to progress with age (Probst *et al.*, 2006; Veltmann *et al.*, 2006). In a large Portuguese family with Brugada syndrome, all 43 family members under age 16 had normal ECGs (Santos *et al.*, 2010). Our relatively small case numbers make other genotype–phenotype or age-related analyses less meaningful, but overall the observations are in keeping with age-related penetrance seen in known inherited cardiac channelopathies.

The Na^+^/K^+^-ATPase transporter is critical in maintaining electrochemical gradients across cell membranes by coupling hydrolysis of ATP with transmembrane 3Na^+^/2K^+^ exchange. The catalytic α-subunit in humans has four isoforms: α_1_, α_2_, α_3_ and α_4_ encoded by *ATP1A1*, *ATP1A2*, *ATP1A3* and *ATP1A4*, respectively, with differential tissue expression. Isoforms α_1_, α_2_, and α_3_ are expressed in the CNS; α_1_ ubiquitously, α_2_ predominantly in astrocytes and α_3_ in peripheral and central neurons; all three isoforms are expressed in healthy human cardiomyocytes with variable mRNA levels of each subunit; 63% (α_1_), 15% (α_2_) and 23% (α_3_) (Zahler *et al.*, 1993). Models of alternating hemiplegia [Myshkin mouse model (Kirshenbaum *et al.*, 2013); *Drosophila* (Ashmore *et al.*, 2009)], together with comparative molecular modelling, have demonstrated that some causal mutations in alternating hemiplegia (p.D801N, p.I274N, p.I810S, p.D923Y) lead to significant structural changes of the ATPase protein, affecting potassium binding and conductance (Ashmore *et al.*, 2009; Kirshenbaum *et al.*, 2013). *In vitro* studies show that p.E815K, p.I274N and p.G947R mutants have loss of ATPase activity and do not bind the ATPase inhibitor, ouabain, compatible with complete loss of function, whereas D801N mutants show absent ATPase activity, but retained ouabain-binding function, indicating abnormal cation binding and reduced K^+^ affinity, lending support to the correlation between E815K and a more severe phenotype (Weigand *et al.*, 2014). The underlying basis of the ECG abnormalities observed is not yet explained, but the findings point to dynamic abnormality of cardiac repolarization reserve. This ‘reserve’ is the physiological redundancy of capacity to repolarize the myocardium that is the result of the multiple inward and outward cardiomyocyte currents that influence repolarization (Roden, 1998). Impaired repolarization reserve is considered important in sudden death associated with inherited cardiac channelopathies, and may possibly have a role in SUDEP.

Our findings suggest that alternating hemiplegia can be considered another cardiocerebral disorder, and that cardiac evaluation, with at least ECG, should be considered in alternating hemiplegia, especially in older (≥16 years) patients. Our data do not permit more specific recommendations, but we note that in some cases, dynamic ECG changes of importance were only seen briefly during prolonged recording. The dynamic nature of ECG changes is reflected in the dynamic nature of many neurological symptoms that is typical of alternating hemiplegia, and may share a mechanistic explanation, though we note that there is obviously no link between the actual timing of ECG and neurological changes. The absence of ECG changes during a seizure or plegic episode does not preclude the existence of ECG changes at other times in the same individual.

We note that the general concept of ‘cardiocerebral channelopathy’ is further underpinned by several recent reports of cardiac arrhythmia, such as long QT syndrome or Brugada syndrome, in single individuals or kindreds with epilepsy due to mutations in ion channel genes such as *KCNH2* (Johnson *et al.*, 2009; Omichi *et al.*, 2010; Zamorano-León *et al.*, 2012; Partemi *et al.*, 2013) and *KCNQ1* (Goldman *et al.*, 2009; de Llano *et al.*, 2015).

Our study has limitations. These include limited sampling of the ECG, leading to possible underestimates of the prevalence of abnormalities; possible referral bias, as invitation to participate followed the publication of a single case report (Novy *et al.*, 2014), though it should be noted that the findings in that case were not typical of those reported here; ascertainment bias is also likely, as patients with alternating hemiplegia who may have been undiagnosed and died early would not have been included, again leading to underestimation of prevalence of abnormalities; and the lack of other functional cardiac data, including echocardiography and measures of cardiac function. ECGs were not reviewed in blinded fashion. Although older patients were more likely to be taking antiepileptic drugs, we show that the use of flunarizine or antiepileptic drugs was not associated with whether a patient had ECG abnormalities or not. Overall, the spectrum of drugs taken is not associated with repolarization abnormalities: interval prolongation (e.g. affecting QTc) and arrhythmias seen with antiepileptic drugs (Surges *et al.*, 2010) were not observed in our sample, while flunarizine has no effect on normal dog heart (Vos *et al.*, 1992). We did not include normal controls, as the waveforms and parameters studied have well-established normal ranges from thousands of individuals (e.g. Rautaharju *et al.*, 2009; Surawicz *et al.*, 2009). The number of cases (five) without *ATP1A3* mutation was small: none of these cases had documented ECG changes. Comparisons between alternating hemiplegia cases and the disease control group remained significant when considering only the *ATP1A3* mutation-bearing alternating hemiplegia cases.

Three-quarters of our cases had had seizures or had a diagnosis of epilepsy (Table 1 and Supplementary Table 1). ECG abnormalities are recognized, and probably under-reported, in epilepsy (Lamberts *et al.*, 2015). Our findings might be considered to reflect the seizure disorders in our patients with epilepsy, but we show that the prevalence both of any abnormality and of repolarization abnormalities is significantly higher in the alternating hemiplegia cases than in an age-matched disease control cohort of people with epilepsy. Moreover, not all patients with ECG abnormalities had epilepsy, and our findings illustrate that in alternating hemiplegia, somatic (cardiac) co-morbidity is not temporally related to plegic episodes or seizures, but probably due to shared expression in heart and brain of mutated protein. In a knock-in mouse model of alternating hemiplegia, with the D801N mutation, there is a higher incidence of sudden death than expected: some mice had witnessed seizure-related death, considered to be SUDEP, but there were also mice ‘found dead’ and others who died ‘spontaneously’ (Hunanyan *et al.*, 2015). Sudden premature death in alternating hemiplegia is not always explained. It has been ascribed to cardiorespiratory dysfunction, for which our findings provide a further basis. Our findings may have broader application to the concept of independent cardiac dysfunction as a mechanism for some cases of sudden death in epilepsy (Parisi *et al.*, 2013), especially with increasing numbers of channels and channel-related pathways being causally implicated in epilepsy. Systematic evaluation of function in organs sharing expression of mutated genes needs consideration with any newly-discovered genetic cause of a condition. In alternating hemiplegia, study of other systems that express *ATP1A3* should also be considered. Systematic longitudinal cardiac studies are also now necessary in alternating hemiplegia, as cardiac arrhythmic death is potentially preventable.

## Supplementary Material

Supplementary Table 2

## Abbreviations

IVCD: intraventricular conduction delay
QTc: corrected QT interval
SUDEP: sudden unexpected death in epilepsy
