# Supplementary material for: Faulty cardiac repolarization reserve in alternating hemiplegia of childhood broadens the phenotype
Source: Brain. 2015 Aug 21;138(10):2859–74. doi: 10.1093/brain/awv243 (PMC4671482; doi:10.1093/brain/awv243)
Supplement: Supplementary Table 2 [file brain_awv243_index.html]

Supplementary Data | Brain

## Supplementary Data

files

- Supplementary Data - jpg file
- Supplementary Data - pdf file
