# Supplementary figures and images for: Faulty cardiac repolarization reserve in alternating hemiplegia of childhood broadens the phenotype
Source: Brain. 2015 Aug 21;138(10):2859–74. doi: 10.1093/brain/awv243 (PMC4671482; doi:10.1093/brain/awv243)

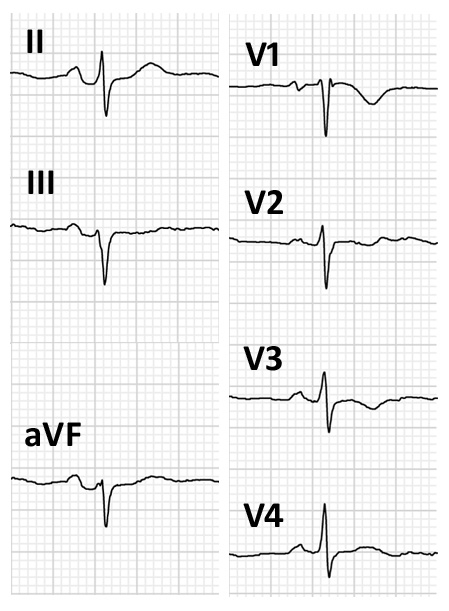

Supplement: Supplementary Table 2 [file 752559eedb7f47b86c77259583f1506f_brain-2014-01943-File012.jpg]
