# Supplementary material for: Faulty cardiac repolarization reserve in alternating hemiplegia of childhood broadens the phenotype
Source: Brain. 2015 Aug 21;138(10):2859–74. doi: 10.1093/brain/awv243 (PMC4671482; doi:10.1093/brain/awv243)
Supplement: Supplementary Table 2 [file Supplementary_material.pdf]

Supplementary Material

Clinical details AH patient cohort

Supplementary Table 1 provides details about epilepsy diagnosis in the AH patient cohort.

Methods

Participants were recruited through the IAHCRC, ENRAH, or personal communication with collaborators, from nine countries. Some participants had whole-exome sequencing as part of previously published studies (Heinzen et al 2012; Rosewich et al 2012). Patients recruited subsequently had *ATP1A3* gene testing by one of two approaches: targeted next-generation gene panel sequencing, or direct Sanger sequencing. Fully anonymised disease control ECGs from patients with epilepsy were obtained from the participating centres (Supplementary Table 2).

Targeted next-generation gene panel sequencing and analysis

Panel design

Five DNA samples from patients with typical AH meeting the clinical diagnostic criteria were sequenced on a neuronal channelopathy-targeted next-generation gene panel as part of a wider study on the genetics of neuronal channelopathies. Amplicons were designed using the Illumina Design Studio software (www.designstudio.illumina.com; Illumina San Diego, CA), to cover the exonic regions of 11 genes implicated in neuronal channelopathies including AH: *CACNA1A, KCNA1, CACNB4, SLC1A3, SLC2A1, ATP1A3, ATP1A2, SCN1A, PRRT2, KCNK18, PNKD* and *PRRT1,* a non-paroxysmal channelopathy gene was on the panel as part of the wider study. A total of 188 amplicons, at a length of 425bp, and cumulative target of 43,834bp were designed. Three exonic regions could not be fully covered: *CACNA1A* (exons 36 and 37); *CACNB4* (exon 2); and *SCN1A* (exon 2). These regions were covered by Sanger sequencing.

Targeted next-generation sequencing and analysis

Genomic DNA (gDNA) previously extracted from whole blood was quantified using the Qubit® 2.0 Fluorometer (Invitrogen, Life Technologies, CA). gDNA at a concentration of 50ng/ul, and DNA absorbance ratio values between 1.8 and 2.0 was used to prepare sequencing libraries using the Illumina TruSeq Custom Amplicon Library preparation kit, according to the manufacturer's protocol (Part # 15027983 Rev. C, Illumina, San Diego, CA). The MiSeq Reagent Kit v3 was used for next-generation sequencing performed on Illumina's MiSeq Desktop Sequencer. Initial data analysis was performed directly on the MiSeq integrated system.

Some samples were sequenced as follows: DNA was fragmented and a next generation sequencing library was prepared using the KAPA Biosystems Library Preparation Kit. A custom designed Nimblegen SeqCap EZ Choice Library was used targeting the protein-coding regions (defined by CCDS) of *ATP1A3*. After enrichment index samples were captured and sequenced using a HiSeq 2000 or 2500 sequencer in the Duke University CHGV-Genomic Analysis Facility. Each base defining the protein coding exons of *ATP1A3* were sequenced at least 10-fold.

Variants were annotated using ANNOVAR (Wang et al. 2010). Rare protein-impacting variants defined as those variants with a minor allele frequency of <1% and with a predicted functional impact (missense, nonsense, in-frame or out-of-frame insertion or deletion, premature stop codon, and splice site variation), as well as novel variants (absent in public databases: dbSNP, Exome Variant Server, and 1000 Genomes cohort) were analysed further. Variants of interest were validated by Sanger sequencing (see below).

Sanger Sequencing

Direct Sanger sequencing of the *ATP1A3* gene was performed in patients recruited after the closure of whole-exome sequencing studies according to a standardized, International AHC Research Group protocol at local centres. A three stage PCR-based sequencing protocol was performed using previously published primers designed for *ATP1A3* (Supplementary Table 3): (i)

sequencing of exons 17 and 18 that are known to carry pathogenic mutations in the majority of AH patients; (ii) if no mutations are identified in exons 17 and 18, sequencing of exons 5,7,8,9,20,21 and 22, known to harbour pathogenic mutations, was performed; and (iii) sequencing of remaining 14 exons in *ATP1A3* was undertaken if no pathogenic mutations were identified in stage (ii). Parental DNA was sequenced if available to establish *de novo* status, or if unavailable, pathogenicity was assigned to the variant only if another patient is reported to carry the same mutation *de novo*.

Supplementary Table 1: Epilepsy in AH patient cohort

Patient/gender	Age of onset of AH (months)	c.DNA change	Amino acid change	Seizures	Epilepsy Diagnosis	Age of onset of seizures	Seizure type(s)	Status epilepticus	AEDs at time of ECG
1 F	0	c.410C>T	p.S137F	+	Generalised	12 wks	GTCS	-	CBZ
2 M	1	c.410C>T	p.S137F	+	Generalised	55 mo	GTCS	+	TPM, MDZ
3 M	0	c.821T>A	p.I274N	-	-	One GTCS at 1 day	GTCS	-	-
4 M	29	c.829G>A	p.E277K	+	-	Single GTCS 29 mo	GTCS	-	-
5 F	18	c.1010T>G	p.L337R	+	Focal	18 mo	Focal and GTCS	-	AZM, PGB, LTG
6 M	3	c.2263G>A	p.G755S	+	Generalised	4 yrs	GTCS	-	TPM
7 M	9	c.2314A>C	p.S772R	+	Generalised	12 mo	GTCS	+	TPM, MDZ
8 M	3	c.2401G>A	p.D801N	-	-	-	-	-	-
9 F	0	c.2401G>A	p.D801N	-	-	-	-	-	VPA, CLB, LZP
10 F	1	c.2401G>A	p.D801N	-	-	-	-	-	-
11 M	17	c.2401G>A	p.D801N	-	-	-	-	-	-
12 M	1	c.2401G>A	p.D801N	+	Generalised	17 yrs	GTCS	-	-
13 F	12	c.2401G>A	p.D801N	+	Generalised	2 mo	GTCS	-	-
14 M	2	c.2401G>A	p.D801N	+	Generalised	8 mo	GTCS	-	-

Patient/gender	Age of onset of AH (months)	c.DNA change	Amino acid change	Seizures	Epilepsy Diagnosis	Age of onset of seizures	Seizure type(s)	Status epilepticus	AEDs at time of ECG
15 M	4	c.2401G>A	p.D801N	-	-	-	-	-	CLZ, TPM, LZP
16 M	2	c.2401G>A	p.D801N	-	-	-	-	-	-
17 F	3	c.2401G>A	p.D801N	⊕	Focal	2 mo	Focal motor	-	-
18 M	0	c.2401G>A	p.D801N	+	Generalised	6 yrs	GTCS	-	LTG
19 F	0	c.2401G>A	p.D801N	⊕	Focal	0	Focal motor, CPS, GTCS	-	TPM, CLZ
20 M	5	c.2401G>A	p.D801N	+	Focal	6 yrs	CPS	-	LEV, TPM
21 F	2	c.2401G>A	p.D801N	+	Focal	10 yrs	CPS, GTCS	-	TPM, CLZ
22 F	4	c.2401G>A	p.D801N	+	Focal	6 mo	Focal	-	KD
23 F	4	c.2401G>A	p.D801N	+	Focal	9 yrs	CPS, GTCS	-	CBZ, TPM
24 F	7	c.2401G>A	p.D801N	+	Focal	5 yrs	CPS, GTCS	-	TPM, CLB
25 F	1	c.2401G>A	p.D801N	+	Focal epilepsy	1 yr 8 mo	Focal seizures	-	VPA, CLB
26 F	1	c.2401G>A	p.D801N	+	Generalised	5 yrs	GTCS	+	VPA
27 F	5	c.2411C>T	p.T804I	-	-	-	-	-	KD
28 M	13	c.2417T>G	p.M806R	+	Generalised	8 mo	Absences, GTCS	-	-
29 F	1	c.2431T>C	p.S811P	+	Generalised	10 yrs	GTCS	-	TPM, PHT, MDZ
30 F	0	c.2443G>A	p.E815K	⊕	Generalised	0	GTCS	-	-
31 M	4	c.2443G>A	p.E815K	+	Focal	19 yrs	CPS	+	ZNS, VPA, LEV, OXC, LCM, CLB
32 M	1.5	c.2443G>A	p.E815K	+	Focal	3 yrs	CPS, GTCS	-	LTG, CLB

Patient/gender	Age of onset of AH (months)	c.DNA change	Amino acid change	Seizures	Epilepsy Diagnosis	Age of onset of seizures	Seizure type(s)	Status epilepticus	AEDs at time of ECG
33 M	1	c.2443G>A	p.E815K	+	Focal	1 mo	Focal, GTCS	-	-
34 F	1	c.2443G>A	p.E815K	+	Generalised	1 mo	GTCS	-	LTG, CLZ, PGB
35 F	1	c.2443G>A	p.E815K	+	Focal	11 mo	CPS, GTCS	-	LEV
36 M	0	c.2443G>A	p.E815K	+	Generalised	2 yrs	GTCS	-	VPA, CLB
37 F	0	c.2443G>A	p.E815K	+	Focal	1 mo	CPS	+	PHT, PGB, CLB, LEV
38 F	6	c.2443G>A	p.E815K	+	Generalised	6 mo	GTCS	+	-
39 M	0	c.2755_2757 delGTC	p.V919del	-	-	-	-	-	-
40 M	1	c.2767G>T	p.D923Y	+	Generalised	1 mo	GTCS	-	VPA
41 M	4	c.2781C>T	p.C927W	+	Generalised	9 yrs	GTCS	+	LTG, CLZ
42 F	1	c.2839G>A	p.G947R	+	Focal	1 mo	Focal seizures	-	CLZ
43 F	1	c.2839G>A	p.G947R	+	Focal	2 mo	Focal seizures	+	CLZ, CBZ
44 F	3	c.2839G>A	p.G947R	-	-	-	-	-	-
45 M	2	c.2839G>A	p.G947R	-	-	-	-	-	-
46 M	0	c.2839G>A	p.G947R	+	NK	NK	NK	-	CBZ
47 M	0	c.2839G>A	p.G947R	+	Focal	6 mo	Focal motor	-	-
48 M	0	No mutation		-	-	-	-	-	-
49 F	4	No mutation		+	Generalised	11 mo	GTCS	-	ZNS, DZP

Patient/gender	Age of onset of AH (months)	c.DNA change	Amino acid change	Seizures	Epilepsy Diagnosis	Age of onset of seizures	Seizure type(s)	Status epilepticus	AEDs at time of ECG
50 F	5	No mutation		-	-	-	-	-	-
51 M	8	No mutation		+	Generalised	8 mo	CPS, GTCS	-	CLB
52 F	7	No mutation		+	Generalised	2 yrs	GTCS	-	-

Key: 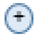 = symptom at onset; + denotes present; - indicates absence; NK = information not known at time of recruitment

AZM = acetazolamide; CAE = childhood absence epilepsy; CBZ = carbamazepine; CLB = clobazam; CGD = Carbohydrate-deficient glycoprotein syndrome, CLZ = clonazepam; CPS = complex partial seizures; DZP = diazepam; ED = emergency department; ESM = ethosuximide; ERP = early repolarisation; g-CSF = granulocyte colony stimulating factor; GTCS = generalised tonic-clonic seizures; hrs= hours; ICU = intensive care unit; IVCD = intraventricular conduction delay; KD = ketogenic diet; LCM = lacosamide; LEV = levetiracetam; LTG = lamotrigine; LZP = lorazepam; MDZ = midazolam; mins = minutes; mo = months; N = no; N/A = not applicable; OXC = oxcarbazepine; P5P = pyridoxal 5’ phosphate; PB = phenobarbitone; PBG = pregabalin; PHT = phenytoin; RAD = right axis deviation; RBBB = right bundle branch block; U = unknown; VGB = vigabatrin; VPA = sodium valproate; wks = weeks; Y= yes; yrs = years; ZNS = Zonisamide

Supplementary Table 2: ECG features, seizure types and medications in matched disease control patients

ID	Age at ECG	ECG findings								Seizure types and/or syndrome	Age of onset of seizures	Drugs at time of ECG	AED response
		Repolarisation abnormality				IVCD	Incomplete RBBB	J wave changes	Other finding on ECG /reason for ECG				
		Anterior	Lateral	Inferior	Widespread								
1	8 mo	-	-	-	-	-	-	-	- / routine in ICU	Epileptic encephalopathy	6 hrs	LEV, TPM, P5P	Y
2	9 mo	-	-	-	-	-	-	-	- / routine for KD initiation	Epileptic encephalopathy	5 mo	CLZ, LEV	N
3	10 mo	-	-	-	-	+*	-	-	- / cardiac evaluation for Ebstein's abnormality	Epileptic encephalopathy	15 mo	None	N/A
4	1 yr 5 mo	-	-	-	-	-	-	-	- / routine	CGD, type unspecified	8 wks	LEV	N
5	2 yrs 4 mo	-	-	-	-	-	-	-	- / routine for KD initiation	Epileptic encephalopathy secondary to polymicrogyria	3 mo	VGB, VPA, KD, MDZ, prednisolone, calcium supplements	N
6	2 yrs 9 mo	-	-	-	-	-	-	-	LAD / routine for KD initiation	Infantile epileptic encephalopathy	6 mo	VGB, LTG, LEV, omeprazole, g-CSF, vitamin D	N
7	2 yrs 10 mo	-	-	-	-	-	-	-	- / routine	Epileptic encephalopathy	1 day	LEV, VPA, VGB	N
8	3 yrs 2 mo	-	-	-	-	+*	-	-	- / routine for KD initiation	Epileptic encephalopathy	3 days	CBZ	N
9	3 yrs 3 mo	-	+	+	-	-	-	-	- / routine for KD initiation	Epileptic encephalopathy secondary to polymicrogyria	3 mo	VGB, VPA, KD, MDZ, calcium supplements	Y
10	3 yrs 11 mo	-	-	-	-	-	-	-	- / routine for KD initiation	Infantile spasms secondary to brain haemorrhage	3 mo	LTG	N

ID	Age at ECG	ECG findings								Seizure types and/or syndrome	Age of onset of seizures	Drugs at time of ECG	Response to AED
		Repolarisation abnormalities				IVCD	Incomplete RBBB	J wave changes	Other finding on ECG/ reason for ECG				
		Anterior	Lateral	Inferior	Widespread								
11	4 yrs 4 mo	-	-	-	-	+*	-	-	- / routine for KD initiation	Progressive myoclonic epilepsy	36 mo	VPA, ESM, LTG	N
12	4 yrs 8 mo	-	-	-	-	-	-	-	- / routine for KD initiation	Hypoxic ischaemic encephalopathy	NK	LEV, KD, selenium, ranitidine, allopurinol, potassium citrate	Y
13	5 yrs 5 mo	-	-	-	-	-	-	-	- / routine	Epileptic encephalopathy	3 mo	PB, VPA	N
14	5 yrs 9 mo	-	-	-	-	-	-	-	- / routine on admission for viral infection	Epileptic encephalopathy	2 wks	CBZ, VPA, VGB, MDZ, ceftriaxone, acyclovir	N
15	6 yrs 9 mo	-	-	-	-	-	-	-	- / routine for KD initiation	CAE	60 mo	VPA, LTG, KD	N
16	7 yrs 3 mo	-	-	-	-	-	-	-	- / routine	Unclassified	41 mo	LEV, LTG, VPA, CLB	N
17	7 yrs 4 mo	-	-	-	+	-	-	-	Borderline QT prolongation / routine for KD initiation	Epilepsy with migrating focal seizures	30 mins of age	PB, VPA, TPM, CLB, KD, MDZ, vitamin D	N
18	8 yrs 3 mo	-	-	-	-	-	-	-	- / routine for KD initiation	CAE	5 yrs	VPA, LTG, KD, calcium supplements, carnitines	Y
19	8 yrs 3 mo	-	-	-	-	-	-	-	- / routine for KD initiation	Epileptic encephalopathy	2 wks	VPA, CLB, MDZ	N

ID	Age at ECG	ECG findings								Seizure types and/or syndrome	Age of onset of seizures	Drugs at time of ECG	Response to AED
		Repolarisation abnormalities				IVCD	Incomplete RBBB	J wave changes	Other finding on ECG/reason for ECG				
		Anterior	Lateral	Inferior	Widespread								
20	8 yrs 7 mo	-	-	-	-	-	-	-	- / routine for KD initiation	Myoclonic epilepsy	4.5 mo	VPA, LEV	N
21	8 yrs 11 mo	-	-	-	-	-	-	-	- / routine for KD initiation	Benign occipital	36 mo	ZNS, STM, MDZ	N
22	9 yrs 3 mo	-	-	-	+	-	-	-	- / routine for KD initiation	Epileptic encephalopathy	4 mo	VPA, LEV, MDZ	N
23	10 yrs	-	-	-	-	+*	-	-	- / routine	Hypoxic ischaemic encephalopathy	22 mo	VPA, CBZ	N
24	10 yrs 9 mo	-	-	-	-	-	-	-	- / routine in ED	JAE	10 yrs	None	N/A
25	11 yrs	-	-	-	-	+*	-	-	- / during respiratory review	Cryptogenic focal	11 yrs	VPA	Y
26	11 yrs	-	-	-	-	-	-	-	- / routine for KD initiation	Early-onset absence	24 mo	VPA, KD, calcium supplements	Y
27	11yrs 10 mo	-	-	-	-	-	-	-	- / routine in emergency department	Frontal	11 yrs	None	N/A
28	13 yrs	-	-	+	-	-	-	-	- / evaluation of syncopal episodes	Rolandic epilepsy	8 yrs	CBZ	Y
29	13 yrs 6 mo	-	-	-	-	-	-	-	- / evaluation for possible cardiogenic syncope	Focal epilepsy due to left hemisphere encephalomal-acia	12 yrs	CBZ	N

ID	Age at ECG	ECG findings								Seizure types and/or syndrome	Age of onset of seizures	Drugs at time of ECG	Response to AED
		Repolarisation abnormalities				IVCD	Incomplete RBBB	J wave changes	Other finding on ECG/reason for ECG				
		Anterior	Lateral	Inferior	Widespread								
30	14 yrs 7 mo	-	-	-	-	+*	-	-	Inferior ERP*/ routine in ED	Genetic generalised	17 yrs 7 mo	None	N/A
31	15 yrs	-	-	-	-	-	-	-	- / routine for KD initiation	Early onset absence	10 mo	VPA, KD	N
32	15 yrs 5 mo	-	-	-	-	-	-	-	- / routine for KD initiation	CAE	9 yrs	KD, LTG, TPM, CLB, Ethinyl-estradiol, levonorgestrel	N
33	19 yrs	-	-	-	-	-	+*	-	- / routine	Cryptogenic focal	4 yrs	TPM, ZNS, CLB	N
34	20 yrs	-	-	-	-	-	+*	-	- / routine	Genetic generalised	14 yrs	VPA, LTG, metformin, olanzapine	N
35	21 yrs	-	-	-	-	-	-	-	- / routine	Genetic generalised	12 yrs	VPA, mirtazapine	N
36	21 yrs	-	-	-	-	-	+*	-	- / routine	Cryptogenic right temporal	15 yrs	VPA, LTG, ZNS	N
37	21 yrs	-	-	-	-	-	+*	-	- / routine	Cryptogenic frontal	11 yrs	LTG, TPM	N
38	24 yrs	-	-	-	-	-	-	-	- / routine	Genetic generalised	14 yrs	VPA, LEV	N
39	24 yrs	-	-	-	-	+*	-	-	- / routine	Symptomatic left occipital	12 yrs	CBZ, PHT, LTG, LCM, ZNS	N
40	25 yrs	-	-	-	-	-	+*	-	- / routine	Cryptogenic right frontal	6 yrs	OXC, LEV, CLB	N
41	25 yrs	-	-	-	-	-	+*	-	- / routine	Cryptogenic temporal	22 yrs	LTG, LEV	N
42	26 yrs	-	-	-	-	-	-	-	- / routine	Cryptogenic focal	13 yrs	LTG	N

ID	Age at ECG	ECG findings								Seizure types and/or syndrome	Age of onset of seizures	Drugs at time of ECG	Response to AED
		Repolarisation abnormalities				IVCD	Incomplete RBBB	J wave changes	Other finding on ECG/reason for ECG				
		Anterior	Lateral	Inferior	Widespread								
43	26 yrs	-	-	-	-	-	-	-	-/ routine	Symptomatic left temporal	3 yrs	LTG, ZNS, CLB, salbutamol	N
44	27 yrs	-	-	-	-	-	-	-	RAD / routine	Symptomatic right hemispheric	9 yrs	LTG, LEV	N
45	27 yrs	-	-	-	-	-	-	-	- / routine	Cryptogenic right frontal	14 yrs	LTG, TPM, CLB, fexofenadine	N
46	28 yrs	-	-	-	+	-	-	-	Inferolateral ERP / routine	Cryptogenic right temporal	12 yrs	CBZ, LCM, TPM, LEV, CLB	N
47	30 yrs	-	-	-	-	-	-	-	- / routine	Multifocal	17 yrs	VPA, CLB	N
48	30 yrs	-	-	-	-	+*	-	-	- / routine	Cryptogenic focal	11 yrs	VPA, OXC, LTG, LEV	N
49	32 yrs	-	-	-	-	-	-	-	- / routine	Symptomatic multifocal	3 yrs	CBZ, PHT, OXC, LCM, AZM, citalopram	N
50	35 yrs	-	-	-	-	+*	-	-	Mild lateral ERP* / routine	Symptomatic right temporal	14 yrs	CBZ, LTG, LEV	N
51	35 yrs	-	-	-	-	-	-	-	- / routine	Symptomatic right hemispheric	4 yrs	VPA, ZNS, PBG, amitryptiline	N
52	35 yrs	-	-	-	-	-	-	-	- / routine	Cryptogenic focal	15 yrs	CLB, simvastatin, almotriptan, citalopram	N

Supplementary Table 2: ECG features, seizure types and medications in matched disease control patients

Key: * normal for age

AZM = acetazolamide; CAE = childhood absence epilepsy; CBZ = carbamazepine; CLB = clobazam; CGD = Carbohydrate-deficient glycoprotein syndrome, CLZ = clonazepam; ED = emergency department; ESM = ethosuximide; ERP = early repolarisation; g-CSF = granulocyte colony stimulating factor; hrs= hours; ICU = intensive care unit; IVCD = intraventricular conduction delay; JAE = juvenile absence epilepsy; KD = ketogenic diet; LAD = left axis deviation; LCM = lacosamide; LEV = levetiracetam; LTG = lamotrigine; MDZ = midazolam; mins = minutes; mo = months; N = no; N/A = not applicable; OXC = oxcarbazepine; P5P = pyridoxal 5' phosphate; PB = phenobarbitone; PBG = pregabalin; PHT = phenytoin; RAD = right axis deviation; RBBB = right bundle branch block; STN = sulthiame; VGB = vigabatrin; VPA = sodium valproate; wks = weeks; Y= yes; yrs = years; ZNS = Zonisamide

Supplementary Table 3: Forward and reverse primers used for Sanger sequencing

Amplification	Forward primer sequence	Reverse Primer Sequence
Exon 1	agaggctcccagcccaag	cccacgaccacatggatt
Exons 2-4	ctcagagacacacagaacca	tgtgacactcactctgggta
Exons 5-6	taaggatctccgaaaggtg	cgacagacccttagattcaa
Exon 7	acccaggcttctagctgtga	tccacacagttggtggaaaa
Exon 8	cacctttcggagatcctta	gagcgtgcacttcttaattt
Exon 9-11	tgtctctgccctgtttctat	ctcccaaagttctggtgtta
Exon 12	acagagcggacaggaatgag	ctttgggcagcatcacaac
Exon 13	gacatagacagagcggacag	ataaaataaaggctgggatg
Exon 14	acttcaccacggacaacctc	tcccagaaagaatgggacag
Exon 15	cccaaagtccttcctcaggt	gtgaggacccaggagtcaag
Exon 16	ttggggacctgaacttcta	ttaaaaagcctccaagtca
Exon 17	agatcgcaccactgcactc	tcttcatgatgtcgctttcg
Exon 18	agcgagactctgtctcaaaa	ctaggccacctaaacatcat
Exon 19	ggcatactcccctctccaag	gatcttacggtgggcagaga
Exon 20	gctgcagtgccactaactga	agagtgagaccctgcctcaa
Exon 21	gcatgtctccccatctctgt	cctggggtcttcggagtaat
Exon 22	tccagtcccctgaaactctg	cggagagatgggaagagaga
Exon 23	cttctcacgggtctctgtctg	cccccagaatacaaaattgg

References

Heinzen EL, Swoboda KJ, Hitomi Y, Gurrieri F, Nicole S, de Vries B, et al. De novo mutations in ATP1A3 cause alternating hemiplegia of childhood. Nat Genet. 2012; 44(9): 1030–4.

Rosewich H, Thiele H, Ohlenbusch A, Maschke U, Altmüller J, Frommolt P, et al. Heterozygous de-novo mutations in ATP1A3 in patients with alternating hemiplegia of childhood: a whole-exome sequencing gene-identification study. Lancet Neurol. 2012; 11(9): 764–73.

Wang K, Li M, and Hakonarson H. ANNOVAR: functional annotation of genetic variants from high-throughput sequencing data. Nucleic Acids Res 2010; 38: e164.
